# A WebGIS platform for the monitoring of Farm Animal Genetic Resources (GENMON)

**DOI:** 10.1371/journal.pone.0176362

**Published:** 2017-04-28

**Authors:** Solange Duruz, Christine Flury, Giona Matasci, Florent Joerin, Ivo Widmer, Stéphane Joost

**Affiliations:** 1 Laboratory of Geographic Information Systems (LASIG), School of Architecture, Civil and Environmental Engineering (ENAC), Ecole Polytechnique Fédérale de Lausanne (EPFL), Lausanne, Switzerland; 2 School of Agricultural, Forest and Food Sciences, Bern University of Applied Sciences, Zollikofen, Switzerland; 3 Institute of Earth Surface Dynamics, Faculty of Geosciences and Environment, University of Lausanne, Lausanne, Switzerland; 4 Institut de Géomatique, Génie d’Environnement et Construction (G2C), Haute-Ecole d’Ingénierie et de Gestion du Canton de Vaud (HEIG-VD), Yverdon-les-Bains, Switzerland; Universita degli Studi di Bologna, ITALY

## Abstract

**Background:**

In 2007, the Food and Agriculture Organization of the United Nations (FAO) initiated the Global plan of action for Farm Animal Genetic Resources (FAnGR). The main goal of this plan is to reduce further loss of genetic diversity in farm animals, so as to protect and promote the diversity of farm animal resources. An important step to reach this goal is to monitor and prioritize endangered breeds in the context of conservation programs.

**Methodology/Web portal implementation:**

The GENMON WebGIS platform is able to monitor FAnGR and to evaluate the degree of endangerment of livestock breeds. The system takes into account pedigree and introgression information, the geographical concentration of animals, the cryo-conservation plan and the sustainability of breeding activities based on socio-economic data as well as present and future land use conditions. A multi-criteria decision tool supports the aggregation of the multi-thematic indices mentioned above using the MACBETH method, which is based on a weighted average using satisfaction thresholds. GENMON is a monitoring tool to reach subjective decisions made by a government agency. It relies on open source software and is available at http://lasigsrv2.epfl.ch/genmon-ch.

**Results/Significance:**

GENMON allows users to upload pedigree-information (animal ID, parents, birthdate, sex, location and introgression) from a specific livestock breed and to define species and/or region-specific weighting parameters and thresholds. The program then completes a pedigree analysis and derives several indices that are used to calculate an integrated score of conservation prioritization for the breeds under investigation. The score can be visualized on a geographic map and allows a fast, intuitive and regional identification of breeds in danger. Appropriate conservation actions and breeding programs can thus be undertaken in order to promote the recovery of the genetic diversity in livestock breeds in need. The use of the platform is illustrated by means of an example based on three local livestock breeds from different species in Switzerland.

## 1 Introduction

### 1.1 Erosion of livestock genetic resources and global strategy for the management of Farm Animal Genetic Resources (FAnGR)

Agricultural biodiversity is the basis of the functioning and the productivity of agricultural systems and is thus essential, for example to satisfy human nutritional needs. Nowadays, agriculture is facing increasing stress due to structural changes and modern farming methods aimed at improving productivity. In the livestock sector, locally adapted breeds have been gradually substituted with a limited number of highly specialized transboundary breeds (such as. the Holstein Friesian cattle selected for milk production) [1] requiring high inputs (e.g. high quality food, medicine) and which are prone to diseases and stress factors which naturally occur [2]. Selective breeding and controlled reproduction of a limited number of high performance individuals have gradually led to a general loss of genetic diversity within breeds [1]. This might reduce productivity through a drop in individual fitness in non-optimal environments, and over the longer term the capacity of the breeds to evolve and adapt to (changing) local environmental conditions (such as climate, pests or diseases) [3].

In order to counteract the current trend of erosion and underutilization of animal genetic resources, the Food and Agriculture Organization of the United Nations (FAO) initiated a global strategy for the management of Farm Animal Genetic Resources (FAnGR) in 2007 [4]. This strategy has been recently reinforced by a recent second report on the state of the world’s animal genetic resources [5]. The ultimate goal of this plan is to lead to policies aimed at promoting and conserving livestock biodiversity and using animal genetic resources in a sustainable way (e.g. priority measures for a sustainable use, development and conservation of animal genetic resources). The FAnGR strategy was discussed and fixed during the Interlaken Conference in 2007 (Interlaken Declaration) and since then, the governments of UN countries are encouraged to implement it. The main objectives of this plan are to identify genetic resources, characterize and protect them in order to stop further genetic erosion and to promote genetic diversity in farm animal resources. An important step to reach this goal is to develop better indicators that can be applied to monitoring genetic trends in domestic populations [6], and to use monitoring systems to identify endangered breeds, to prioritize them, and to initiate as well as support conservation programs [7].

### 1.2 A multi-criteria approach

One of the major challenges is the definition of meaningful criteria to identify endangered breeds. FAO created a scale of endangerment based on the number of breeding females and males [8]; this approach has the advantage of being easily implemented but is a simplistic view of the problem. Several other systems to categorize endangered livestock breeds have been developed on national [9–12] and international levels [13–18]. With a few exceptions [10,12,18], existing systems rarely provide a standardized definition and measurement of the most significant factors [10].

In accordance with the FAO Global Plan, the top strategic priority is given to the characterization of animal genetic resources (AnGR), to the monitoring of trends and risks to these resources, and to the establishment of breed endangerment Early Warning Systems (EWS) [4]. In order to obtain an overview of the diversity, status and trends of animal genetic resources, the measurement of genetic diversity is a basic component of monitoring systems. However, beside genetic diversity, other criteria should also be considered. For instance, Alderson [19] specifies that introgression—the process of uncontrolled entrance of genes from another gene pool through mating with another breed [20]—is another important and relevant criterion since it dilutes specific traits that might be worth conserving. Moreover, the UK monitoring system accounts for geographical concentration of the breeds, as much as a breed that is clustered in a small region is more vulnerable to epidemics [19]. In addition, the presence of cryo-conserved gametes is an important element to consider, as it can refresh genetic resources of very small breed [21] and even help to bring a breed back to life even after a critical point has been reached [22]. Finally, according to the FAO protocol, the supervision of the genetic diversity should be completed by the establishment of national sustainable use policies taking into account environmental and socio-economic aspects, including demographic changes, climate change, and conducting economic and cultural valuation (Strategic Priority 3 [4]).

### 1.3 Data integration: Geographic information system (GIS) and multi-criteria decision analysis (MCDA)

Animal genetic resources have to be monitored and conserved at local, regional and global levels [4] and thus geography is an important component in this effort. Intriguingly, despite the issue of AnGR conservation being composed of entities which are totally embedded in lands and distributed over territories, geography is only considered in the UK system [10]. However, this monitoring system does not assess the level of sustainability of regional or local breeding conditions, comprised of socio-economic, socio-demographic, and environmental characteristics.

Here we propose the application of a GIS-based multi-criteria analysis for the integration of multi-disciplinary data in order to monitor animal genetic resources at different scales. Geographic Information Systems (GIS) offer an appropriate basis in a monitoring perspective as it integrates different categories of information (demography, phenotypes, husbandry practices, socio-economy, natural environment, etc.) on different geographical scales (local, regional or global) [23]. Furthermore, GIS analysis exhibits other advantages such as a direct comparison between available thematic layers according to geographic coordinates, and the production of valuable outputs like maps, graphs, and tables) [24].

Whether combined with the use of GIS or not, multi-criteria decision analysis (MCDA) has often been applied to support decisions involving environmental issues and biodiversity conservation [12,25–27]. However, the application of GIS-based multi-criteria analysis are rare in the domain of livestock species conservation.

In parallel, when dealing with multi-criteria analysis, we are often confronted to the problem of non-commensurability, which occurs in situations where criteria are assessed onto different and incomparable scales of measure [28]. This difficulty is often circumvented by means of mathematical tools. Within this type of approach, we find many methods, including the Multi-Attribute Utility Theory (MAUT) [29] or the Analytic Hierarchy Process (AHP) [30] methods, as well as the use of weighted average methods. However, criteria are not necessarily comparable, and this is the reason why methods referred to as “outranking methods” were introduced, giving the possibility that two scenarios might be incomparable [31,32]. This situation is recurrently encountered when dealing with FAnGR evaluation, in which criteria cover a wide variety of fields measured with different units, from pedigree information to socio-economic data. Nevertheless, outranking methods also present drawbacks, such as the difficulty in explaining and implementing them as well as a reduced performance when the number of variants is very large. These are the reasons why here we consider the MACBETH method [33], which is a weighted average method with satisfaction thresholds defined by experts in the disciplines considered (see section 2.3).

### 1.4 GENMON: A WebGIS platform to monitor breed endangerment

To cope with the challenge of the identification of endangered breeds, we propose an easy-to-use WebGIS platform (GENMON), designed to facilitate decision-making which should favor sustainable use and conservation of livestock breeds via the integration of five important categories of information: pedigree analysis, introgression, geographical concentration, cryo-conserved material and agriculture sustainability (this being calculated on the basis of socio-economic and environmental data). Investigated breeds are then ranked in order to identify the most endangered ones using weighting of the various criteria. The GENMON application has been designed in the Swiss context and uses data available in this country; however, the system can easily be adapted to the data available in other countries.

The usefulness of the GENMON approach is demonstrated here through its application to three local livestock breeds, the Swiss Original Braunvieh cattle (OBV), the Valais Blacknose sheep (VBN) and the Franches-Montagnes horse (FM). The OBV is from central Switzerland, not ranked among endangered breeds but under supervision because of its international interest due to valuable genetic heritage [34]. The VBN is a sheep breed mainly reared in Valais, which is recognized for its genetic uniqueness [35,36]. The FM horse breed is the only Swiss native horse breed [37] and is mostly bred in the Jura mountains. For reproducibility reasons, a fourth breed has been simulated (SIM), whose pedigree has been made publically available (see section 3.1.2)

## 2 Materials and methods

**Fig 1:**
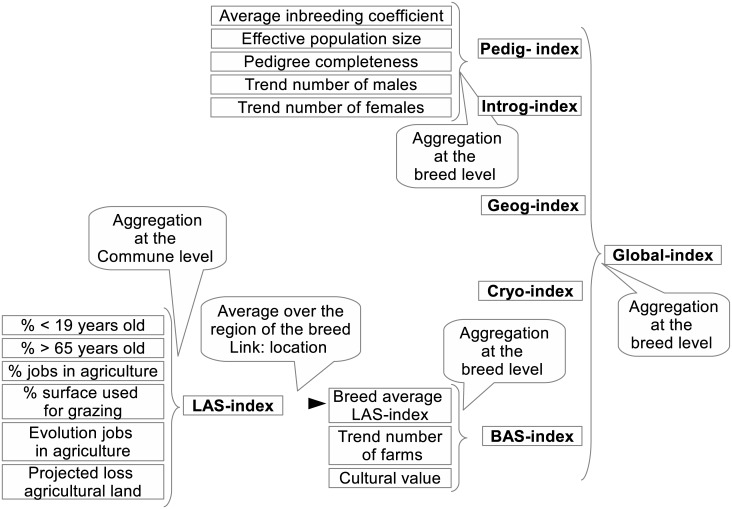
Simplified overview of the GENMON Process. GENMON takes into account five main categories (or indices) aggregated into one final score: pedigree information (pedig-index); introgression (introg); geographic distribution (geog); cryo conservation plan (cryo); socio-economic and environmental information (BAS, standing for breed agriculture sustainability). Some of these indices come from an aggregation of criteria themselves.

**Fig 2:**
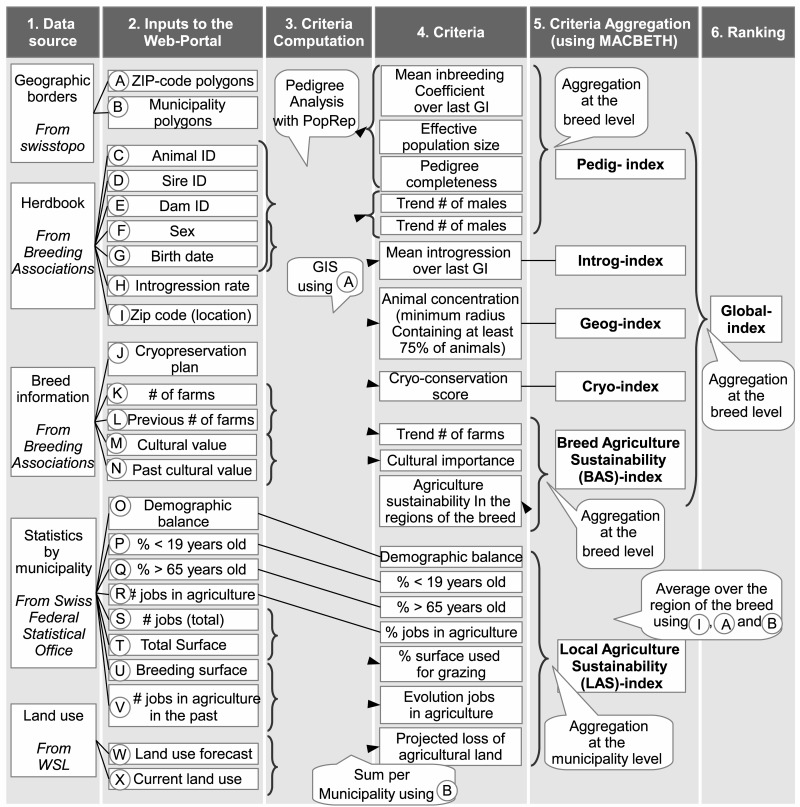
Overall GENMON Process. The process starts with data input followed by criteria processing, integration and aggregation; GI: generation interval, GIS: Geographic Information System, Pedig-Index: index accounting for pedigree and genetic diversity, Introg-Index: introgression index, LAS/BAS Index: Local/Breed Agriculture Sustainability indices, accounting for socio-economic and environmental sustainability of breeding conditions; swisstopo is the Swiss Federal Office of Topography (http://www.swisstopo.admin.ch/, WSL is the Swiss Federal Institute for Forest, Snow and Landscape Research.

The process implemented in GENMON is based on multiple stages and its overview is represented in Fig 1, whereas a more detailed description is given in Fig 2. GENMON relies on the aggregation of indices (pedigree information, introgression, geographic distribution, cryo conservation plan and socio-economic and environmental information) into one final score (Fig 1). The process as a whole (Fig 2) begins with the upload of different sources onto the WebGIS portal. These data are geographic borders (ZIP-codes and municipality areas), herdbook information (pedigree data), general information on the breed (such as cryo-conservation or cultural value), municipality-based socio-economic and environmental data, and land use change scenarios. A set of criteria is obtained by slightly modifying the inputs (such as the trend of the number of farms being computed out of current and past number of farms; see Fig 2 for more examples), and are then aggregated at the breed and at the municipality levels before the ranking based on a global index of sustainability characterizing breeds can be processed.

### 2.1 Data

In this section we list and describe the different input data (see Fig 2, input column), their source and how they are pre-processed to extract the information for the different criteria.

#### 2.1.1 Geodata: Swiss municipalities and ZIP-codes

The GENMON application has been developed to integrate different categories of information in order to monitor animal genetic resources. All categories have a geographical component and the GIS-based multi-criteria analysis platform developed allows the different data sources to be linked and compared according to geographic coordinates (concept of spatial coincidence). Thus geographical units are key to this platform. Here we use a shapefile containing the geographic coordinates of Swiss ZIP-code areas corresponding to the situation in June 2013 [38] as defined by Swiss Post (http://www.post.ch/). With this file, the animals to be geo-referenced according to the unique identifier (ZIP-code) of the mail delivery area where they are bred. The shapefile contains 4,191 polygons covering the whole country. In parallel, a shapefile with 2,564 municipalities (2013) is used [39] to geo-reference the socio-economic data characterizing the territory, which is not available at the ZIP-code level. The link between socio-economic data and their corresponding polygons is made through the unique identifier of municipalities as defined by Swiss Statistics (www.statistics.admin.ch/).

#### 2.1.2 Animal and breed information

The data used to characterize investigated animals are summarized in Table 1. They include pedigree information (dam, sire, sex, birthdate, and introgression) as well as the ZIP-code where the animal was born. The standard format is given in the third column (Type), but the user also has the option of specifying the format of the data if it does not meet the standard format (see remark in the fourth column).

**Table 1:** Description of the variables to be provided from the breeding organizations at the individual level.

Parameter	Input	Type	Remark
C	Animal ID	Text	
D	Sire ID	Text	
E	Dam ID	Text	
F	Sex	M/F	Or as specified
G	Year of birth	Eg. 2009	The whole date can also be specified
H	Introgression	Real [0;1]	Or Real [0;100]
I	ZIP code	e.g. 3096	

From Table 1, the animal, sire and dam ID as well as the sex and birthdate (parameters C to G) are used to run the pedigree analysis with the PopRep module [40]. This analysis calculates the mean inbreeding coefficient by year, the generation interval and the effective population size. The introgression of each animal (input H) is used to compute the mean introgression over the last generation interval.

The ZIP-code (input I) is used for two purposes. On the one hand it makes it possible to link animals with socio-economic variables at their respective locations, and on the other hand it enables the determination of the geographical concentration of breeds (see section Geographical concentration). This parameter is computed with the help of spatial SQL (structured query language). Given the lack of a precise position for each individual, the centroid of the ZIP-code polygons containing the animals is used as an approximation.

The introgression rate of each animal corresponds to the fraction of animals from other breeds in the pedigree. This individual rate is used to calculate the average introgression rate of the breed over the last generation interval. GENMON requires the introgression rate of each animal to be computed before being uploaded onto the database.

The Swiss Original Braunvieh data were provided by the Swiss Brown Cattle Breeders' Federation [41] and consisted of a pedigree file containing 94,099 animals born between 1923 and 2014 (56% during the last decade). For the Franches-Montagnes breed, data were made available by the Swiss Federation of the Franches-Montagnes horses [42] and the herdbook included 46,166 animals, born between 1831 and 2013, (67% during the last decade). For the Valais Blacknose, data were produced by Swiss sheep breeders organization [43] and contained 110,584 sheep born between 1910 and 2012 (86% during the last 10 years). The fourth simulated breed (SIM) was obtained from an existing pedigree in which we changed IDs, birthdates, introgression rates and geographic distribution of all animals. Some individuals have been removed while others have been shuffled in the pedigree. The final pedigree contained 65,664 simulated animals born between 1920 and 2016 (63% during the last ten years). Though this breed does not represent any specific species, it was entered in the system as a pig breed. Therefore, weights and thresholds are assigned to it as if it were a pig breed. The pedigree is available at http://doi.org/10.5281/zenodo.220887

Additionally, the breeding associations are also asked to provide general information about the breeding activities listed in Table 2.

**Table 2:** Description of the variables required to characterize the breed to be monitored, provided by the breeding association.

	Input	Type	Remark
J	Cryo-conservation management plan	Presence of frozen semen (yes/no) and of a real cryo-conservation management plan (yes/no)	
K	Number of farms	Integer	To compute the trend of the number of farms
L	Number of farms 5 years ago	Integer	
M	Cultural value	Does the breed have a cultural value (yes/no)	To compute the cultural value score
N	Past cultural value	Did the cultural value of the breed decrease in the recent past (yes/no)	

The information on the cryo-conservation (input J in Table 2) is used to compute the Cryo-index (see column criteria aggregation of Fig 2).

The current and past number of farms (input K and L) are used to compute the trend of the number of farms, while the current and past cultural value (input M and N) will give a cultural value score (0 if it has no cultural value, 0.5 if the value does exist but is decreasing and 1 if the value exists and is stable) and all four inputs are used in the BAS index. Cultural value include criteria such as antiquity, role of the agricultural system, farming techniques, role in landscape, gastronomy, folklore and artistic expression [44]

#### 2.1.3 Socio-economic and environmental data over the Swiss territory

Data used for the socio-economic and environmental characterization of breeding locations are summarized in Table 3. They include statistics on demographic facts (demographic balance, percentage of young and old people), the importance of agriculture (number of jobs in agriculture compared to the total number of jobs, the surface used for animal breeding compared to the total surface of the commune, past number of jobs in agriculture) as well as current and forecasted land use.

**Table 3:** Data input and characteristics for socio-economic and environmental assessment.

	Input	Type	Remark
O	Increase/decrease in population over the last 2 years	%	
P	Percentage of the population younger than 19 years	%	
Q	Percentage of the population older than 65 years	%	
R	Number of jobs in the primary sector		To compute the percentage of jobs in the primary sector
S	Total number of jobs (all three sectors)		
T	Total surface of the commune	km^2^	To compute the percentage of grazing surface
U	Surface used for animal breeding	ha	
V	The number of jobs in the primary sector from a previous year		To compute the evolution of jobs in the primary sector considering two years
W/X	Current land use and land use forecast		WSL

Unless specified in the “Remark” column, these variables were obtained from Swiss Statistics (http://www.bfs.admin.ch/bfs/portal/en/index.html).

Socio-economic data used for the analyses (input O to V in Table 3) are provided by Swiss Statistics: they were extracted from the regional portraits of municipalities for the year 2014 [45] except the surface used for animal breeding (input U) which comes from STAT-TAB (2012), a dynamic interface specifically designed to download data from the Swiss Statistics Office[46] as well as the previous number of jobs in the primary sector (input V) which was extracted from STAT-TAB (2010).

Land use GIS-layers (W and X) [47] were also used to calculate the percentage of agricultural land that would be lost in the future, which gives an estimation of future regional sustainability of agriculture. In their original format, they consist of six files (current state of land use plus five scenarios for 2050) containing ASCII grids with cells of 1 ha spatial resolution. Projections according to five scenarios are available. Some of them are based on different policies as described by IPCC [48]. With the intent of facilitating the use of GENMON, only one scenario (the “trend” scenario”) is taken into account. It is calculated using a linear interpolation of the total area of each land use type based on the land statistics of 1985, 1997 and 2009. This scenario seems to be a reasonable assumption, given that unless very strict policies are set up, the trend that has been noticed over the last few decades is likely to continue. A comparison between the present and future states is carried out outputting a percentage of agricultural land loss per ZIP-code.

Note that the Local Agriculture Sustainability (LAS) Index is calculated for all municipalities (including those without breeding activities). A description of how the information at the municipality level is linked to information at the breed level is given in the section Multi-criteria aggregation.

#### 2.1.4 Integration of geographic data

**Fig 3:**
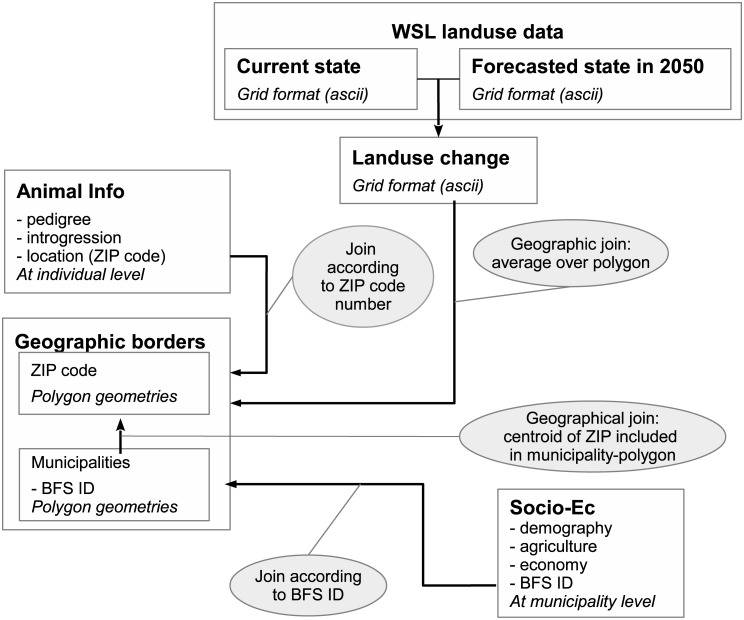
Link between the different geographic data types (point, polygons and grids). All components are brought to the ZIP code level. The links are done either by joining attributes (ZIP code number, BFS ID) or according to geometries. BFS ID: unique identifier from the statistical office.

The data input described above shows various geometry types. Therefore, a succinct description of the method used to match the different spatial units is proposed in Fig 3. All components are brought to the ZIP code level: The links are done either by joining attributes (ZIP code number, commune unique identifier) or according to geometries.

### 2.2 Selection of relevant criteria

In this section we describe the criteria included in GENMON in order to quantify aspects that are important to evaluate and monitor the conservation status of the breeds under study. They belong to five categories: 1) pedigree-related issues and genetic diversity, 2) introgression, 3) geographical concentration 4) cryo-conservation plan and 5) agriculture sustainability. The selection of criteria has been discussed with a group of 12 experts, scientists and professionals whose activities are related to livestock breeding and management, within a workshop organized for this purpose. The size of the group is justified by the need to favor discussion and emergence of ideas between experts. The description of the discussion course is made available in the supporting information (S1 Appendix).

#### 2.2.1 Pedigree analysis

Pedigree information can be used to analyze the genetic structure of respective populations [40]. Since in most countries (including Switzerland) DNA-sampling in livestock, and especially in local livestock breeds, is not currently performed on a regular basis and is not available for marker-based genetic analysis, pedigree data is a valuable alternative to approximate genetic diversity within populations [49]. Many researchers have already investigated the problem of pedigree analysis and there are several software solutions that can be used for this purpose (e.g. PopRep [40]). In pedigree-based methods, the inbreeding rate can be estimated from pedigree data, which is then used to estimate the effective population size (Ne). The inbreeding coefficient (*F*) is a measure of the relatedness of ancestors [50], and the effective population size (Ne) is the number of breeding individuals in an idealized population (see for example [16,51]). The effective population size is an essential parameter in conservation genetics and population management because of its direct relationship with the level of inbreeding, fitness and the amount of genetic variation loss in populations [52]. In consequence, these two parameters (*F* and Ne) are calculated and taken into consideration as criteria in GENMON. Given that the inbreeding coefficient is computed at the individual level, it is necessary to perform an average of this coefficient. In order to alleviate problems arising from missing or incomplete pedigree data in certain years, here we compute the average inbreeding coefficient over the last Generation Interval (GI, average age of parents when giving birth to their offspring [40]), as an alternative to the average the last year for which data is collected. Nevertheless, this choice has the disadvantage of reducing the relative importance of recent data resulting in a greater inertia of the coefficient, which makes it more difficult to identify sudden changes in the pedigree structure. Admittedly, the inbreeding coefficient has known drawbacks in specific situations (e.g. after a bottleneck, the inbreeding coefficient will not decrease even when the effective population size rises), but we decided to retain this coefficient, as it is a value that breeders are familiar with. Breeder’s involvement into the process is essential to successfully undertake the whole GENMON process.

As regards the effective population size (Ne), several methods have been proposed and output different values with the same data [53]. This is the reason why GENMON proposes four different Ne values based either on the evolution of inbreeding or on coancestry (see [40] for more details). Poprep calculates these values for every year, each time using animals in the last generation interval. Only the value of the last year is taken into account in the computation of the pedig-index, corresponding in fact to the last generation interval.

We also included the pedigree completeness as a criterion to maximize, in order to counterbalance the fact that breeds with incomplete pedigree will artificially achieve a low level of inbreeding. While it is true that such breeds are not necessarily endangered, GENMON will lower their final score to emphasize potential problems concealed by their incomplete pedigree. This criterion is computed as the average pedigree completeness at the sixth generation (output by poprep) over the last generation interval. It is necessary to take deep pedigree completeness because the first generations are usually almost 100% complete, which does not discriminate between complete versus incomplete pedigree.

In parallel to the PopRep analysis, the trend of the number of females and males over the last five years is also computed, giving an insight into the evolution of the breeding practices.

#### 2.2.2 Introgression

The introgression rate is entered by the user for each animal. It corresponds to the percentage of foreign blood (based on the fraction of ancestors in the pedigree belonging to other breeds) per individual. Like the inbreeding coefficient, the average computation takes place over the last generation interval.

#### 2.2.3 Geographical concentration

To quantify the geographical concentration of breeds, Alderson [19] proposed to compute the smallest circle containing at least 75% of the animals, centered on the centroid of animal positions.

#### 2.2.4 Cryo-conservation plan

The presence of a cryo-conservation plan is also taken into account via a score bounded between 0 and 1. If such plan exists and follows the FAO guidelines for the given specie [54], the score for the breed is 1. Conversely, if the material to be frozen are collected in an uncontrolled manner, the score is lowered to 0.5. Indeed, if the individuals from which gametes will be collected are not chosen with care, close kinship between selected animals can possibly exists and the value of the cryo-conservation is lessened [55]. Ultimately, if no gametes are cryo-conserved, the score is 0.

#### 2.2.5 Breed agriculture sustainability

To calculate the agricultural sustainability for each breed, we take two components into account: statistics on regions where the animals are reared (which leads to a local agriculture sustainability index) and general breed information. The first component, i.e. local agricultural sustainability index (i.e. a score quantifying how sustainable agriculture is in a given municipality), is assessed by an approach inspired by Bertaglia *et al*. [27] who quantified the marginality of a region (i.e. areas where possible land uses are relatively limited) combining land use, demographic and socio-economic data using a deliberative approach for variables selection. Here we replaced this deliberative step by a representative panel of experts (see Fadlaoui *et al*. [56]) with complementary skills (ecology, animal production, agricultural science, socio-demography). The goal is to assess sustainability, represented by the three constituent parts of sustainable development (social, economic and environmental [57]) and to include at best information directly characterizing agriculture and breeding activities. On this basis, a series of discussions between the 12 experts (see S1 Appendix) involved in the present research resulted in the selection of seven variables. Three variables are related to the socio-economic situation of the municipalities: demographic balance, percentage of inhabitants younger than 19 years and older than 65 years respectively, while the other variables account for rural and farming-related features describing municipalities: percentage of active people in the primary sector, percentage of surface used for breeding activities, employment trend in agriculture in the past years (we used data from 2010 compared to the situation in 2012) and projected agricultural land loss (the percentage of agricultural land lost by 2050, as calculated by Price *et al*. [47]). The underlying concept represented by each variable is explained in the next paragraph.

Regions with a too negative demographic balance (i.e. demographic changes through natural change of population and migration) are often marginal regions where cattle breeding activities can be difficult to pursue [27]. Negative consequences are expected for breeders and farmers of the municipality since, if a trend of depopulation exists, it is likely that the region will be subject to a lack of manpower. Indeed, on top of the important problem represented by heirs (i.e. manpower) abandoning the family farm, regions facing man power shortage are usually economically not attractive and economic activities (including agriculture) will generally decrease. Regarding the age structure, a municipality with a high proportion of older and retired inhabitants will face financial problems in the future if there is no migration of professionally active younger people. This could lead to a tax increase in order that municipalities are still able to pay the costs for infrastructure, pensions or for hospital care. Indeed, Alderson [19] proposed to directly use the age of the breeder as a criterion, which would be a valuable alternative in our case. However, this parameter is difficult to evaluate and the corresponding data is not available in Switzerland. Then, the percentage of farmers provides a trustable insight into the predisposition of the municipality for activities in the primary sector. In fact, such an indicator reveals the available manpower existing in a given region, a crucial condition for the establishment and preservation of farming activities. Furthermore, a high percentage of farmers in a municipality is a sign of well-developed agricultural practices. This is an indication that the sustainability of breeding activities is likely to be ensured in such regions. Similarly, the percentage of surface used for cattle breeding activities suggests which regions of Switzerland are suited for this kind of activities. Municipalities with a large number of cattle farms will be those where long-term sustainability is higher.

As regards the criterion used to translate employment trend in agriculture, the difference of the percentage of people working in agriculture among years (here between 2010 and 2012) informs about current employment dynamics in this field of activity. Finally, as a last criterion, the agricultural land loss projection from Swiss Federal Institute for Forest, Snow and Landscape Research (WSL) for 2050 is used [47]. This is a relevant criterion to assess the sustainability of breeding activities. Two kinds of land losses are mainly considered: agricultural land abandonment with subsequent forest growing as well as urban sprawl. Besides, the projections also take into account the consequences of the predicted climate change.

The second component at the breed level assesses breeding practices including the evolution in time of the number of farms in which animals are reared. This criterion adds another relevant dimension to the criterion considering the trend of the number of animals (described in section Pedigree Analysis). Indeed, if a large number of animals are still alive but are reared in a sole farm, the breed can be considered as endangered, since the future of the breed practically depends on the decisions of a single person. Additionally, the cultural value of the breed and its evolution is also considered, as by definition a breed being culturally significant will be better sustained by local people, so as to prevent its extinction [4].

In the following section, we explain how these two components (evolution of number of farms and of cultural values of the breed) are aggregated and how they relate to other criteria.

### 2.3 Multi-criteria aggregation

**Fig 4:**
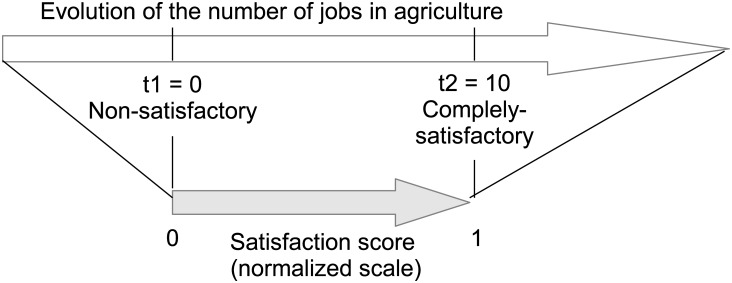
Criterion scaling with the MACABETH method for the variable “Evolution of the number of jobs in agriculture”. Evolution of the number of jobs in agriculture represents the difference of the number of jobs in agriculture between 2010 and 2012 (in %); the upper arrow indicates the initial values while the lower arrow represents the values that have been normalized between 0 and 1 (*s*_*j*_). The satisfaction thresholds for this criterion is defined as being 0 and 10%. The values of the satisfaction thresholds are given in S1 Appendix.

This section describes the process of criteria aggregation, necessary to allow comparison among breeds. As stated in the introduction, several methods exist to handle the problem of integrating various criteria, and the one chosen here is the MACBETH method, which is a weighted average using satisfaction thresholds [33]. Using this approach, decision-makers have the possibility to establish the importance (weight) and a minimum and a maximum expected value for all criteria considered. For each criterion x_*j*_ (Fig 1 lists which criteria are included in which (sub)-index), we defined a null satisfaction threshold t*n*_*j*_ and a complete satisfaction threshold t*c*_*j*_ (S1 Appendix). Then, the values of the municipalities for all criteria falling within the defined range have been linearly scaled between 0% satisfaction and 100% satisfaction (0 and 1 respectively), while scores exceeding the limits have been bound to these minimal and maximal threshold values. A partial satisfaction score *s*_*j*_ per criterion is therefore obtained. Eq 1 synthesizes the transformation while Fig 4 illustrates the scaling procedure applied to one of the variables.

$$s_{j}=\;\{\begin{array}{ll} 0 & if\;x_{j}<\;tn_{j}\\ 1 & if\;x_{j}>\;tc_{j}\\ \frac{x_{j}-\;tn_{j}}{tc_{j}-\;tn_{j}} & otherwise \end{array}$$ (1)

The approach results in the attenuation of extreme values since we cut the tails of the distribution of the variables.

In a successive step, a weighted sum of the scaled scores *s*_*j*_ using a weight *w*_*j*_ associated with each of the criteria has been performed, providing a global satisfaction percentage *S* for each municipality, ranging from 0% to 100% and interpreted as a sustainability index (Eq 2).

$$S=\sum_{j=1}^{m}w_{j}s_{j}\;where\;\sum_{j=1}^{m}w_{j}=1$$ (2)

As shown in Figs 1 and 2, the MACBETH method is used at two levels to compute: 1) the sub-indices (fifth column in Fig 2: criteria aggregation (sub-indices); e.g. Pedig-index) containing themselves several criteria (i.e. inbreeding coefficient and effective population size for the Pedig-index) and 2) the global-index (sixth column: ranking based on the global index). With the purpose to decide where to place satisfaction thresholds and weights and to validate these choices, a spatial and statistical exploratory analysis of the different criteria has been carried out as a preliminary step (S2 Appendix). Subsequently and on the basis of the preliminary analysis, satisfaction thresholds and weights were defined for each variable based on the opinion of the 12 experts involved (the chosen values of the weights and thresholds are given in S1 Appendix).

A special note is required as regards the Breed Agriculture Sustainability index. Indeed, it contains statistical components produced at the municipality level to calculate the Local Agriculture Sustainability Index, LAS, and information produced at the breed level. Once the LAS is computed over all municipalities, a mean over the regions where investigated animals are reared is calculated (weighted by the number of animals per region). This value is then aggregated (according to the MACBETH method) with the criteria at the breed level (evolution of the number of farms, and the cultural value). This aggregated parameter is then named “Breed Agriculture Sustainability” (BAS) Index. This score gives an insight into the sustainability of breeding conditions for a specific breed.

### 2.4 Web-portal implementation

In this section we describe the technology used to develop the GENMON application while we will argue the reasons supporting these choices in the discussion section. All technologies presented here are open-source.

The main part of the interface is built in Hypertext Markup Language (HTML) and Hypertext Preprocessor (PHP) language. The upload of a file (containing either animal information or socio-economic variables) is made through an HTML-form. The file is then stored in a database management system (DBMS). Here we use PostgreSQL [58] with its spatial extension PostGIS [59]. Once stored in the database, the pedigree analysis is completed by PopRep [40]. PopRep is a software coded in Perl, which performs a pedigree analysis, stores the results in the PostgreSQL database and produces three output reports about population structure, inbreeding and population size. Then, Structured Query Language (SQL)-queries are executed to aggregate values: averages and sums over the last generation interval for the whole breed as well as per ZIP-code. The indices (Pedig-, Introg-, Geog-, Cryo-, LAS-, BAS- and the Global-index) are computed. The weights and thresholds used for this computation must be provided by the user via the interface before the upload and are stored in the database. The whole procedure is described in the tutorial section of the interface.

For the visualization part, the interface is built on OpenLayers [60], a javascript library. The layer of the map is stored in PostGIS and published to the web with the use of Geoserver [61]. The map is displayed as a Web Mapping Service (WMS) image and exhibits the attributes of the polygons as a choropleth map (for example the variable “mean inbreeding” of the municipality). To obtain more information on a given selected municipality (additional attributes), a WFS (Web Feature Service) request is run. This enables the display of statistics for a region, as for example the total number of animals in the selected zone or the mean inbreeding.

## 3 Results

In this section, we give an overview of the outputs produced by GENMON, illustrated by means of three Swiss breeds: the Valais Blacknose sheep (VBN), the Franches-Montagnes horse (FM) and the Swiss Original Braunvieh cattle (OBV).

### 3.1 Summary table

**Fig 5:**
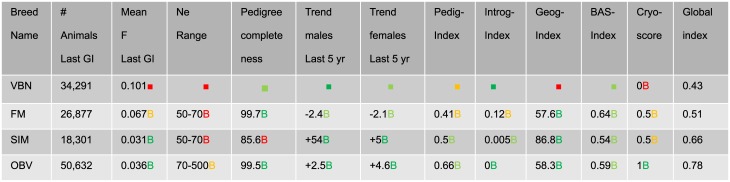
Summary output table of the GENMON application. The global index and its different components for three Swiss breeds. The colors give the degree of satisfaction of each criterion (red: not satisfactory, dark green: totally satisfactory). The breeds are ordered from the most threatened on top to the healthiest at the bottom. (GI: generation interval, F: inbreeding coefficient, Ne: population size. The description of the indices is given in section 2; VBN: Valais Blacknose sheep, FM Franches-Montagnes horse, OBV Swiss Original Braunvieh cattle). The colors are assigned according to the following thresholds (expressed in satisfaction score): 10% is the limit between red and yellow; 50% defines the limit between yellow and light-green; 95% corresponds to the threshold between light and dark green.

The main output of GENMON is a summary table enabling the comparison between breeds according to the different criteria mentioned above, which are then aggregated in a global index (see Fig 5). Each line displays the summary information of one breed, whereas each column represents each criterion individually and the global index. To facilitate a comprehension at first glance of endangerment status of all breeds under study, breeds are ranked according their global scores (most threatened on top). Furthermore, a color code for each criterion gives the level of satisfaction according to the thresholds defined by the user (red: not satisfactory, green: satisfactory).

Fig 5 shows that according to the weights and thresholds that were applied, the VBN is the most endangered breed with a relatively low global index (0.36), while the OBV seems to be a healthy breed (global index = 0.69). Beside these observations, the specific problems of each breed can also be quickly identified from the sub-indices. For example the VBN suffers from a high mean inbreeding coefficient (mean F = 0.101) and is spatially very concentrated (Geog-index = 12.9). On the other hand, the FM is significantly introgressed (Introg-Index = 0.12), which lowers its global index. The SIM breed has an incomplete pedigree which lowers its pedig-index and finally its global score. It has to be noted that weights and thresholds of the pedig-index are set differently for each species (see S1 Appendix). We propose here across-species early warning but the user can easily separate species and rank the breeds accordingly.

### 3.2 Detailed investigations

**Fig 6:**
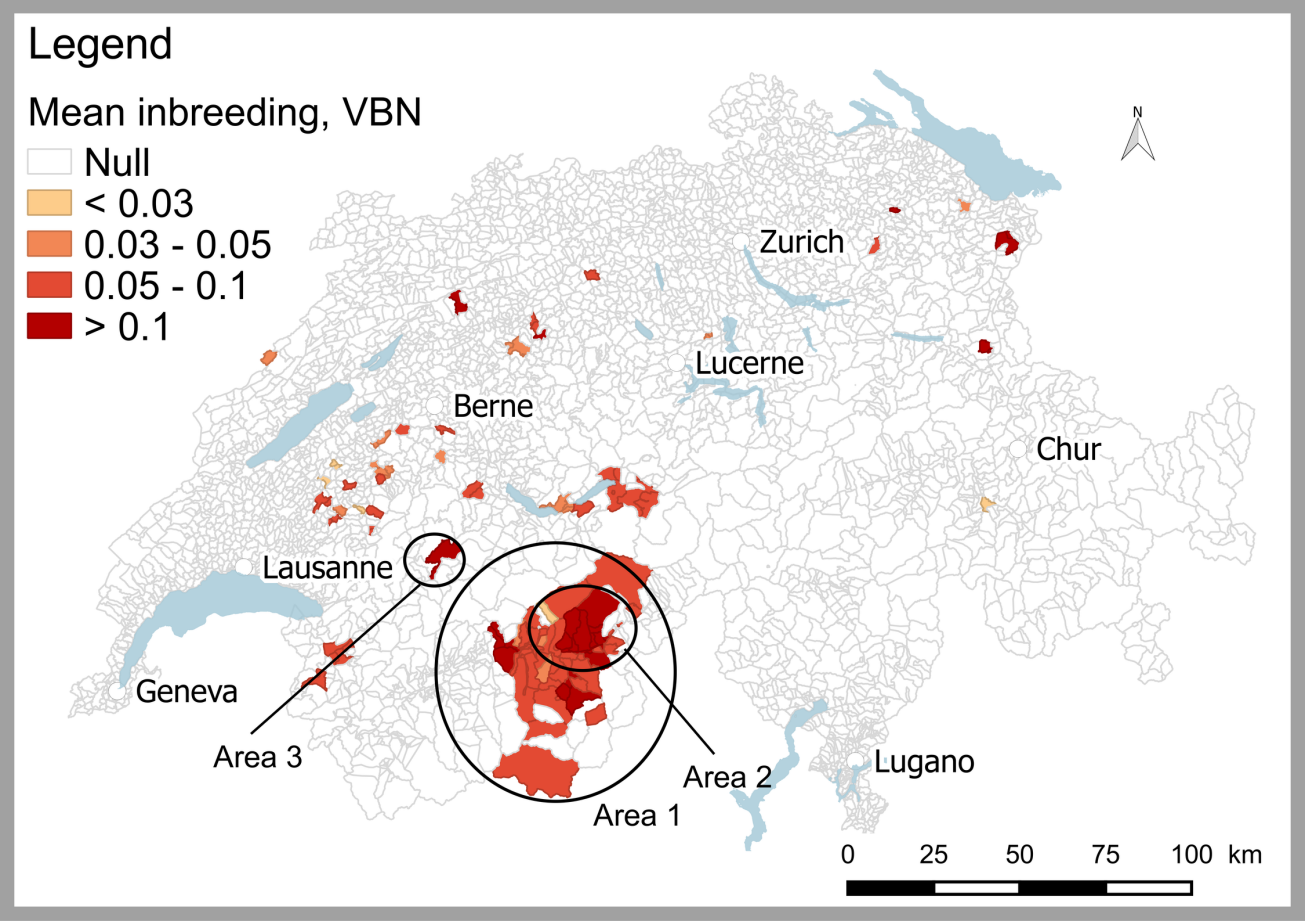
The geographical distribution of inbreeding coefficients per ZIP-code for the Valais Blacknose (VBN) sheep. The mean inbreeding is computed over the last generation interval (2010–2012).

**Fig 7:**
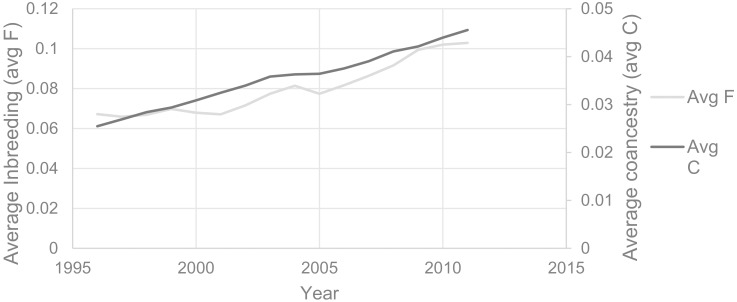
Inbreeding and coancestry coefficient for the Valais Blacknose (VBN) sheep breed between 1994 and 2012. Progression of more than 0.04 of the inbreeding and 0.02 of the coancestry in 15 years; the current average inbreeding is remarkable and exceeds 0.1 while the coancestry exceeds 0.04.

Once the main problems are identified, more detailed information can be obtained on the breeds of interest. Here we show the details for the breed with the lowest global index, namely the VBN; it is of interest to visualize the geographical distribution of inbreeding coefficients (Fig 6) and its evolution over the last few years (Fig 7).

The VBN is very concentrated in a small portion of the Swiss territory (in facts 98% of animals are located within area 1 of Fig 6). The mean inbreeding coefficient is higher than 0.05 in almost all regions and some ZIP-codes area exceed 0.1. More precisely, 61% of the municipalities with VBN show a mean inbreeding higher than 0.03 (value that has been chosen for the satisfaction threshold) and 4% have a mean inbreeding higher than 0.1 (the value corresponding to the non-satisfaction threshold). Geographical distribution maps are essential to identify specific regions with high inbreeding coefficients that would require a more intense assistance in breed management (typically area 2 of Fig 6). Furthermore, to better identify regions which are critical for certain breeds and in order to assess the importance of a region for the breed, distribution maps of inbreeding coefficients can be compared with maps showing the number of animals per municipality, (see S1 Fig). This also allows the user to identify regions with only few animals, in which the inbreeding coefficient is likely to be overestimated (e.g. area 3 of Fig 6). With GENMON, other maps can be calculated as for example the geographical distribution of the introgression rate, shown here for the FM breed (S5 Fig).

### 3.3 Local agriculture sustainability index

**Fig 8:**
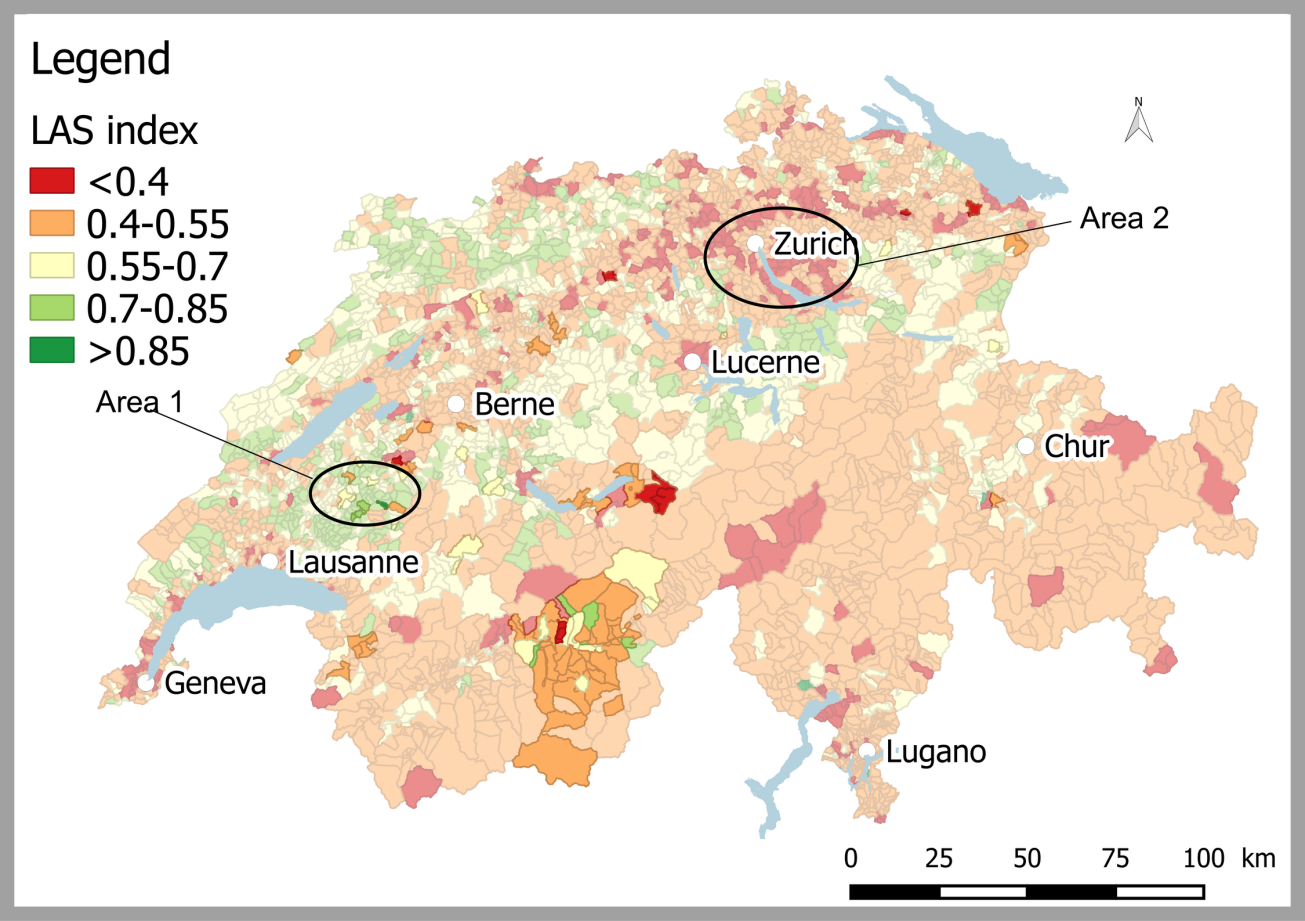
Geographical distribution of the local agriculture sustainability (LAS) index. The computation based on weights and thresholds described in S1 Appendix. The colors of the areas that do not contain any Valais Blacknose sheep are faded. Sustainable areas are shown in green (e.g. area 1) while low sustainability is represented in red (e.g. area 2 situated in an urban low-land zone). The pale yellow shows intermediate values.

The spatial distribution of the local sustainability index, allowing a ranking of all Swiss municipalities (including those having no animals uploaded in the GENMON application) is shown in Fig 8. This cartographic representation of the sustainability index integrates social, demographic, economic and environmental characteristics of the different regions of Switzerland at the municipality level.

The Swiss territory shows different regional sustainability trends. Low sustainability values are found mainly in the Plateau region located between Lausanne, Berne and Zurich (Fig 8).

The best conditions for breeding activities are found primarily in lowland/mid-altitude areas located in the region between the Plateau and Jura (Fig 8). These municipalities are highlighted in green on the map (Fig 8). Actually, the States of Vaud and Fribourg (area 1 in Fig 8) as well as the central Switzerland have many regions with relatively high sustainability scores, potentially favorable for farm animal breeding activities. The Jura Mountains as well show high sustainability values, mainly in the States of Neuchâtel and of Jura. In order to identify if a specific breed is reared in sustainable conditions, the “Breed Agriculture Sustainability” index (BAS-index, Fig 1) can be used, which computes the average over the regions where animals are reared and weighted by the number of animals per region. In this case, the majority of municipalities with VBN are classified in the categories where the farming activity have low sustainability scores (only 48% of the ZIP-code areas containing VBN have a LAS index larger than 0.55).

## 4 Discussion

### 4.1 Performance of the GENMON application

GENMON offers a ranking evaluation of the level of endangerments based on a straightforward application. This service has not been implemented anywhere else and might serve as a good basis in order to initiate, support and supervise prioritization and conservation programs as required by the FAO protocol [4]. Being straightforward and easy-to-use, the application can be applied to a large number of breeds. Consequently, the Federal Office for Agriculture in Switzerland (BLW) intend to use GENMON to monitor local breeds in the future.

The use of georeferenced animal data as proposed in GENMON is also unique in FAnGR. Indeed, the English system of livestock endangerment scale only uses geography to assess geographic concentration of breeds. The integration of different data type such as socio-economic and environmental factors is made possible using georeferenced data that is often ignored in livestock conservation. The idea of assessing sustainability of breeding condition has already been proposed [27], but has not been implemented in an automated pipeline until now.

Both in the English system and in GENMON, the estimation of the geographic concentration is a rough approximation. It is mainly used to assess the ability of diseases to spread between flocks, but does not take the barriers or paths of the environment into account. A better approach could be inspired by the assessment of the geographic range and the area of occupancy in wildlife conservation (see for example [62]).

GENMON also offers flexibility, since the weights and thresholds can be adjusted, which enables an adequate modelling of the situation depending on the species and the country. The selection of variables, weights and threshold parameters used to build the LAS and BAS sustainability indices is part of a participatory process involving experts, in order to select the proper social, demographic and economic factors affecting the development of specific livestock breeds. Care must be taken when deciding the weights and thresholds. Indeed these parameters will have a considerable influence on the final output. Here we undoubtedly face a heuristic problem with unknown properties, so that we have no way of assessing how good the final score is. As a result, the panel of experts must be representative and diverse enough to represent different backgrounds, breeding associations and professional activities related to the livestock sector. Indeed its role is to select a robust set of parameters translating the policy the government agency wants to apply. A key role will be played by specialists of the surveyed breeds to fine-tune these parameters. In the case we present here, the selection of parameters and the tuning of weights was carried out by a panel of experts working for the Swiss Federal Office for Agriculture (FOAG), and involved in the sector of animal production responsible for the monitoring of livestock breeds.

When confronted to GENMON, breeders reacted positively and actively gave their opinions to improve the application. They expressed their satisfaction in being able to step back and discover in more detail the context of breed monitoring that considers various criteria, to evaluate the status of their breed when compared to other and to diagnose which components of their breed could be improved.

GENMON has been designed for the specific case of Switzerland and relies on the data availability of this country. Nonetheless, the methodology could be used in other places, with inevitable modifications to adapt to the accessible data. For example, it is not mandatory to use ZIP codes, but one could use other administrative divisions. In addition, DNA instead of pedigree could be used to assess the level of inbreeding. The weights and thresholds should be discussed in each country individually, depending on the specific environmental conditions, breeding practices, policy implemented and data available.

Finally, GENMON quickly assesses the conservation status of breeds and to identify and prioritize vulnerable ones. Moreover, a rapid identification of factors affecting the conservation of breeds is possible through the detailed results (e.g. for the VBN Figs 6 and 7). This might serve as a good basis in order to initiate, support and supervise prioritization and conservation programs.

### 4.2 Technology chosen

An important technological challenge was to use open source software only to develop GENMON. Indeed, open source technology have increased transparency due to the availability of code, offers a greater flexibility, given that source code can be modified according to the need of the application. [63]. Open source technology will also favor the implementation of this solution in other countries.

It has been chosen to build GENMON as a Web-service rather than a desktop application, so that a unique and central database will collect and store information coming from different sources. Moreover, given that the computations carried out with GENMON are intensive (especially the pedigree analysis), it is of interest to perform the computation on the server-side, which frees the user’s computer for other tasks.

With regards to the software used in GENMON, the DBMS PostgreSQL was chosen since it is one of the most efficient open source DBMS (notably due to its capacity to handle georeferenced data efficiently [64]) and because it easily communicates with an interface built in PHP. Moreover, the PopRep code used for the pedigree analysis uses a PostgreSQL database, which facilitates the data transfer. For the pedigree analysis, PopRep has been favored over other pedigree software like CFC [65] or ENDOG [66] because it has already been successfully used by FOAG, and people from this institution are familiar with its outputs. Furthermore, PopRep also has the advantage of directly creating detailed reports, which can be useful for further analyses. Openlayers [60] has been selected for the cartographic environment, for it offers a large flexibility and is well documented.

## 5 Conclusions

GENMON was developed to satisfy requirements of the “Global Plan of Action for Farm Animal Genetic Resources” launched by FAO, which still needs to be set up in many countries. This application has been developed as a useful tool for FAnGR monitoring that could assist many countries in this task, provided they have sufficient data (including pedigree and socio-economic variables).

It is an easy-to-use WebGIS application relying on open source software solutions and provides a multi-criteria approach for monitoring endangered breeds based on subjective thresholds of a government agency. By means of geographic coordinates, the application integrates different types of criteria, including pedigree data, genetic introgression, socio-economic and environmental aspects and geographical concentration of the breeds under study to evaluate the local context in which they are reared. GENMON computes a global sustainability index for each breed, making it possible to compare the endangerment level of several species and/or breeds, while enabling the identification of the most important problems and their geographical location for breeds separately, on the basis of sub-indices. Based on these outputs, a detailed examination of the conservation status of breeds can be carried out, which might serve as a firm basis for proposing prioritization policies. An important contribution of GENMON is to provide decision-makers with a clear identification of breeds, municipalities and corresponding breeders that should be supported with special policies in order to maintain a lively and sustainable breeding sector.

The GENMON application will be expanded with new features. A relevant example is the potential future use of genetic data following the methodology described by vanRaden *et al*. [67], so as to complete pedigree information if it is not complete or to replace it to avoid the time-consuming pedigree analysis step and to assess inbreeding and effective population size in particular. However, current system developments will soon make it possible to process conservation indices based on marker-based genetic information as well.

The GENMON application is functional and can be accessed using the following link: lasigsrv2.epfl.ch/genmon-ch. A sample file is available for users interested in testing the upload of a file. The code is available on GitHub: https://github.com/SolangeD/GENMON.

## Supporting information

S1 Appendix Description of the workshop procedure to obtain thresholds and weights. (PDF)

S2 Appendix Descriptive statistics of the selected variables used in the local agriculture sustainability index. (PDF)

S1 Fig The geographical distribution of the number of individuals per ZIP-code for the Valais Blacknose (VBN) sheep. (TIF)

S2 Fig The geographical distribution of mean inbreeding coefficients per ZIP-code for the Original Braunvieh (OBV) cattle. (TIF)

S3 Fig The geographical distribution of the number of individuals per ZIP-code for the Original Braunvieh (OBV) cattle. (TIF)

S4 Fig The geographical distribution of mean inbreeding coefficients per ZIP-code for the Franches-Montagnes (FM) horse. (TIF)

S5 Fig The geographical distribution of introgression per ZIP-code for the Franches-Montagnes (FM) horse. (TIF)

S6 Fig The geographical distribution of the number of individuals per ZIP-code for the Franches-Montagnes (FM) horse. (TIF)
