# Supplementary material for: A WebGIS platform for the monitoring of Farm Animal Genetic Resources (GENMON)
Source: PLoS One. 2017 Apr 28;12(4):e0176362. doi: 10.1371/journal.pone.0176362 (PMC5408993; doi:10.1371/journal.pone.0176362)
Supplement: S1 Appendix — (PDF) [file pone.0176362.s001.pdf]

S1 Appendix

Description of the workshop procedure to obtain thresholds and weights

12 people participated to the workshop, which were scientists and professionals whose activities are related to livestock breeding and management. In Switzerland, decisions related to Farm Animal Genetic Resources are taken by a group constituted of 10-15 experts working for the Federal Office for Agriculture (FOAG). Here - as GENMON is funded by FOAG - we adopted the same operational mode and involved the same people. The panel of experts includes people from academia (4), breeding associations (4), government agencies (2) and livestock industry (2). The participants received one week in advance the questionnaire posted at the end of this appendix, in order to enable a better preparation to feed the discussion.

On the day of the workshop, the application was first described. Then the participants were asked to fill in the sub-mentioned questionnaire on thresholds and weights. The answers were synthetized during a break, and a discussion among participants followed, to reach a consensus.

In the end, the following weights and thresholds were retained (table S1).The list of criteria is slightly different from the one given in the questionnaire, as some criteria were removed while other were added:

Table S1: Weights and thresholds retained after the workshop.

Index	*Criteria*	*weight*	*Threshold T1*	*Threshold T2*
Global index	Pedig-Index	50		
	Introgression	15	15%	3%
	Geographic concentration	15	20km	50km
	BAS - Index	10		
	Cryo-conservation	10		
Pedig-Index	Mean inbreeding	15	10% (15% for pigs)	3% (5% for pigs)
	Effective population size	40	50	250
	Pedigree completeness	15	87	97
	Trend males (last 5 years)	15	-5%	0%
	Trend females (last 5 years)	15	-5%	0%
BAS-Index	Demographic balance	5	0	3
	% jobs in agriculture	10	1	16
	Evolution of jobs in agriculture (%)	10	0	10
	% areas for breeding	15	6	30
	% of predicted agricultural land change	20	94	100
	% >65 years	15	20	4
	% <18years	5	3	10
	Cultural value of the breed	10		
	Evolution of the number of farms	10		

The full description of criteria is given in the Materials and Methods section. The weights and thresholds of the pedig-index can be set differently for each species. However, the experts decided that only pigs should have different thresholds.

GenMon Workshop

Criteria, weights and thresholds values

Your name (optional): ______________________

GenMon relies on different criteria, weighting factors for their consideration within the (sub-)indices. Additionally thresholds have to be defined to indicate the users (breeding organisations, NGOs and public bodies) if action is required or not. The goal of this work is to define relevant criteria, weights and thresholds with experienced persons involved in the management of AnGR of Switzerland for the further development of GenMon.

Therefore we would be grateful if you could fill in the following form during the 30 next minutes. We will then collect these documents and compile the information provided so that after the lunch break we can discuss the outcome together, based on your comments, and elaborate a consolidated list of criteria with related weights and thresholds.

The table at the end of the document summarizes all pieces of information for which we would like you to give your opinion. Please read first the instructions to understand how to fill in this table.

- **Criteria**

  The upper mentioned table and the figure 1 below lists all of the 14 criteria we are considering so far. If you have any new criteria to consider in mind, please write them down in the empty lines at the end of the table. On the other hand, if a criterion does not make sense to you, please indicate that in the comment column.

- ***Weighting***:

  Weights are used to confer the relative importance of each criterion. The system works like this: let us assume you have 100 points to distribute among the different criteria; you will attribute more points to the most important criteria.

  Here we have to define the weight of the components of the final index (1 in the figure hereunder) and of two sub-indices (2 and 3 in the figure). In total you have 300 points to distribute (100 per index or sub-index).

  If you think that a criterion does not make sense, you can assign it a weight of 0. In that case, please indicate in the comments why you think so.

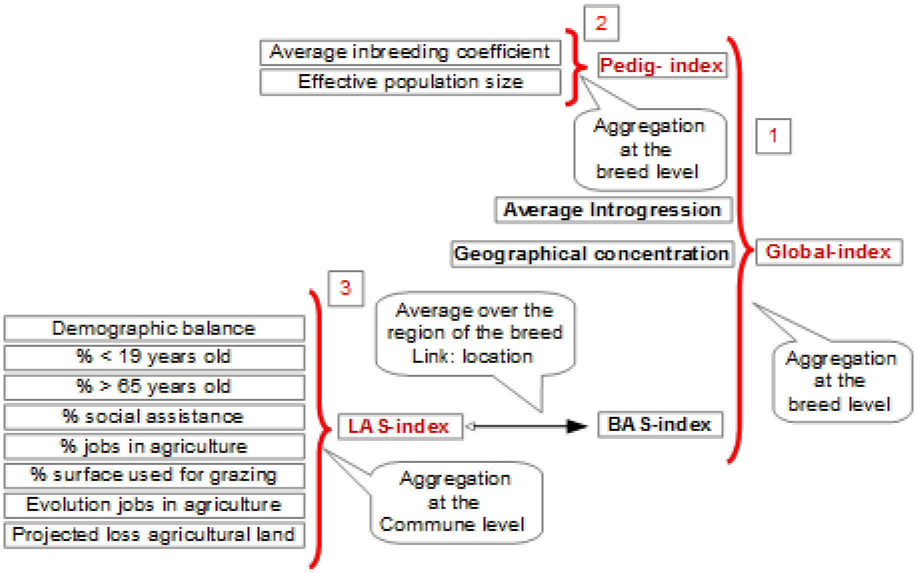

Figure 1: Criteria and their aggregation

- ***Thresholds (T1 and T2)*:**

For each of the following criteria, we have to define a non-satisfaction (T1) and a satisfaction threshold (T2). In the context of monitoring the thresholds will indicate if immediate action is required or not. Thereby the thresholds are the levels of the different criterions where the lights of the "monitoring-ample" are turning from red (not acceptable) to orange or to green (totally acceptable). It means that you have to decide for each criterion what is the lower limit value corresponding to a non-satisfactory situation, and what is the upper limit value corresponding to a satisfactory situation. The following figure provides an example of how thresholds work. This example is the criterion "average inbreeding". Here the satisfaction threshold is defined to 2% change, and any percentage below 2% will get the maximum satisfaction score (1=100% satisfactory). On the other hand, the non-satisfaction threshold is set to 10% and any breed having an average inbreeding higher than 10% have the lowest satisfaction score (0=0% satisfactory).

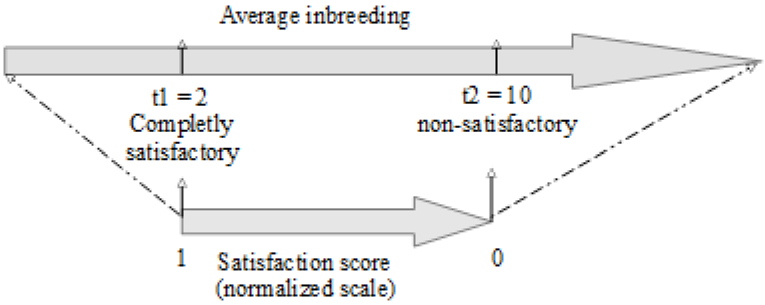

Figure 2: Scaling of a criterion using satisfaction thresholds

Note that there is no need to fill in gray cells in the table.

Moreover, if some thresholds apply only to specific species, please mention it in the comments column. You can also provide different thresholds for different species if you want (indicate it clearly in the comments column)

To help you determine the thresholds, we provide you with some ranges, explanations and statistics

- Introgression and Inbreeding: ranges between 0 and 100 %
- Geographic spread: This corresponds to a radius containing 75% of the animals. As an example, the Schwarznasenschaf has a radius of 13km, while the Braunvieh original has a radius of 59km.
- Agriculture sustainability: some statistics about Swiss communes are given in the table below. Also, the chosen non-statisfaction (T1) and satisfaction (T2) thresholds. Therefore, only indicate those thresholds (light gray in the table to fill in) if you do not agree with the proposed thresholds.

	Min	Max	Average	Standard deviation	Chosen T1	Chosen T2
Demographic balance	-18.2	38.8	1.8	3.4	0	3
Social assistance rate	0	11.4	1.9	1.7	5	2
% jobs in agriculture	0	100	15.8	16.1	1	16
Evolution of jobs in agriculture (%)	-100	1300	9.4	37.0	0	10
% areas for breeding	0	100	24.1	17.9	6	30
% of predicted agricultural land kept	78.5	100	98.4	2.5	94	100
% pop >65 years	0	36.4	21.2	3.5	20	4
% pop <18years	6.3	66.7	17.7	4.1	3	10

- **General comments?**

  If you want to give a general comment, you are encouraged to do so at the back of the page.

index	*Criteria*	*Weight*	*T1*	*T2*	*Comments*
Global 1	Genetic/pedigree (inbreeding, Ne)				
	Introgression				
	Geographic spread				
	Socio-economic criteria				
Genetics/ pedigr[illegible] 2	Inbreeding				
	Population size				
Agriculture sustainability 3	Demographic balance		0	3	
	Social assistance rate		5	2	
	% jobs in agriculture		1	16	
	Evolution of jobs in agriculture (%)		0	10	
	% areas for breeding		6	30	
	% of predicted agricultural land kept		94	100	
	% pop >65 years		20	4	
	% pop <18years		3	10	
New criteria?					

General comments
