# Supplementary material for: A WebGIS platform for the monitoring of Farm Animal Genetic Resources (GENMON)
Source: PLoS One. 2017 Apr 28;12(4):e0176362. doi: 10.1371/journal.pone.0176362 (PMC5408993; doi:10.1371/journal.pone.0176362)
Supplement: S2 Appendix — (PDF) [file pone.0176362.s002.pdf]

S2 Appendix

Descriptive statistics of the selected variables used in the local agriculture sustainability index

The summary statistics of the 7 variables as well as the non-satisfaction (t1) and satisfaction thresholds (t2) applied in the context of this paper are displayed in table S2.

Table S2: Description of the variables used in the LAS-index

	Min	Max	Average	Standard deviation	t1	t2
% Landuse forecast 2050	78.5	100	98.4	2.5	94	100
Demogr. Balance	-18.2	38.8	1.8	3.4	0	3
Job agriculture	0	100	15.8	16.1	1	16
Grazing surface	0	100	24.1	17.9	6	30
Young	0	36.4	21.2	3.5	3	10
Old	6.3	66.7	17.7	4.1	20	4
Evolution jobs	-100	1300	2.6	37.0	0	10

t1 indicates the non-satisfaction threshold, t2 the total satisfaction threshold. Landuse forecast: percentage of agricultural land still used for agriculture in 2050; Demogr balance: difference in population between 2010 and 2012; Job agriculture: percentage of jobs in primary sector; grazing surface: the percentage of surface used for breeding activities; Young: percentage of people younger than 19 years; Old: percentage of people older than 65 years; Evolution jobs: difference in number of jobs in primary sector between 2010 and 2012)

The variables shown in table S2 have been subjectively selected by a group of 12 scientists as a priori significant and meaningful. The chosen t_{nj} and t_{cj} limits are always selected in the vicinity of the 1st and, respectively, the 3rd quartiles of the distributions.

For the demographic balance the null satisfaction was set to 0, i. e. all the municipalities having experienced a population decrease between 2012 and 2014 receive a satisfaction score of 0%. Complete satisfaction was obtained if the population of a municipality increase of 3%, a proportion deemed to be sufficiently large to bring concrete benefits.

The proportion of farmers deemed to yield null satisfaction has been set to 1% of the active population, a threshold avoiding municipalities with an underdeveloped agricultural sector to receive excessively bad satisfaction score. A symmetric logic is applied to the upper tail of the distribution, with rural municipalities exceeding 16% reaching a complete satisfaction level of 100%.

Then, for the percentage of surface used for breeding activities, 30% of grazing surface in a municipality was deemed sufficient to achieve the maximal satisfaction, whereas values below 6% were recognized as equally unsatisfying.

Finally, as regards the evolution of the number of jobs in agriculture, the null satisfaction was set to 0, so that municipality losing jobs in agriculture have a 0 satisfaction score. On the other hand, a positive evolution was judged completely adequate if it exceeded 10 full-time equivalents jobs.

The data range existing between null and complete satisfaction thresholds is comparable between the different variables: it always corresponds to the same partial satisfaction interval (0% to 100%).

Independence of selected variables

Table S3 shows the correlation matrix between the 8 variables selected, computed for the 2,564 Swiss municipalities. Selected criteria show a satisfactory independence with a largest correlation (in absolute value) of -0.67 between the percentage of people younger than 19 years old and those older than 65. This correlation was expected and the weights accorded to these two variables should be set accordingly, in order not to overweight the age structure of the population. All other correlation values are quite low (the second largest value being 0.41), which translates a sufficient level of non-redundant information for a proper assessment of the sustainability of the breeding activities in the Swiss municipalities.

Table S3 : Correlation matrix between the 8 variables included in the LAS-index

	Landuse forecast	Demogr. Balance	Job agriculture	Grazing surface	Young	Old	Evolution jobs
Landuse forecast		-0.10	0.19	-0.31	-0.13	0.29	0.06
Demogr. Balance			-0.15	0.00	0.19	-0.29	0.02
Job agriculture				0.29	0.20	0.05	-0.08
Grazing surface					0.41	-0.31	-0.17
Young						-0.67	-0.09
Old							0.05
Evolution jobs							

Landuse forecast: percentage of agricultural land still used for agriculture in 2050; Demogr balance: difference in population between 2010 and 2012; Job agriculture: percentage of jobs in primary sector; grazing surface: the percentage of surface used for breeding activities; Young: percentage of people younger than 19 years; Old: percentage of people older than 65 years; Evolution jobs: difference in number of jobs in primary sector between 2010 and 2012
