# Supplementary figures and images for: A WebGIS platform for the monitoring of Farm Animal Genetic Resources (GENMON)
Source: PLoS One. 2017 Apr 28;12(4):e0176362. doi: 10.1371/journal.pone.0176362 (PMC5408993; doi:10.1371/journal.pone.0176362)

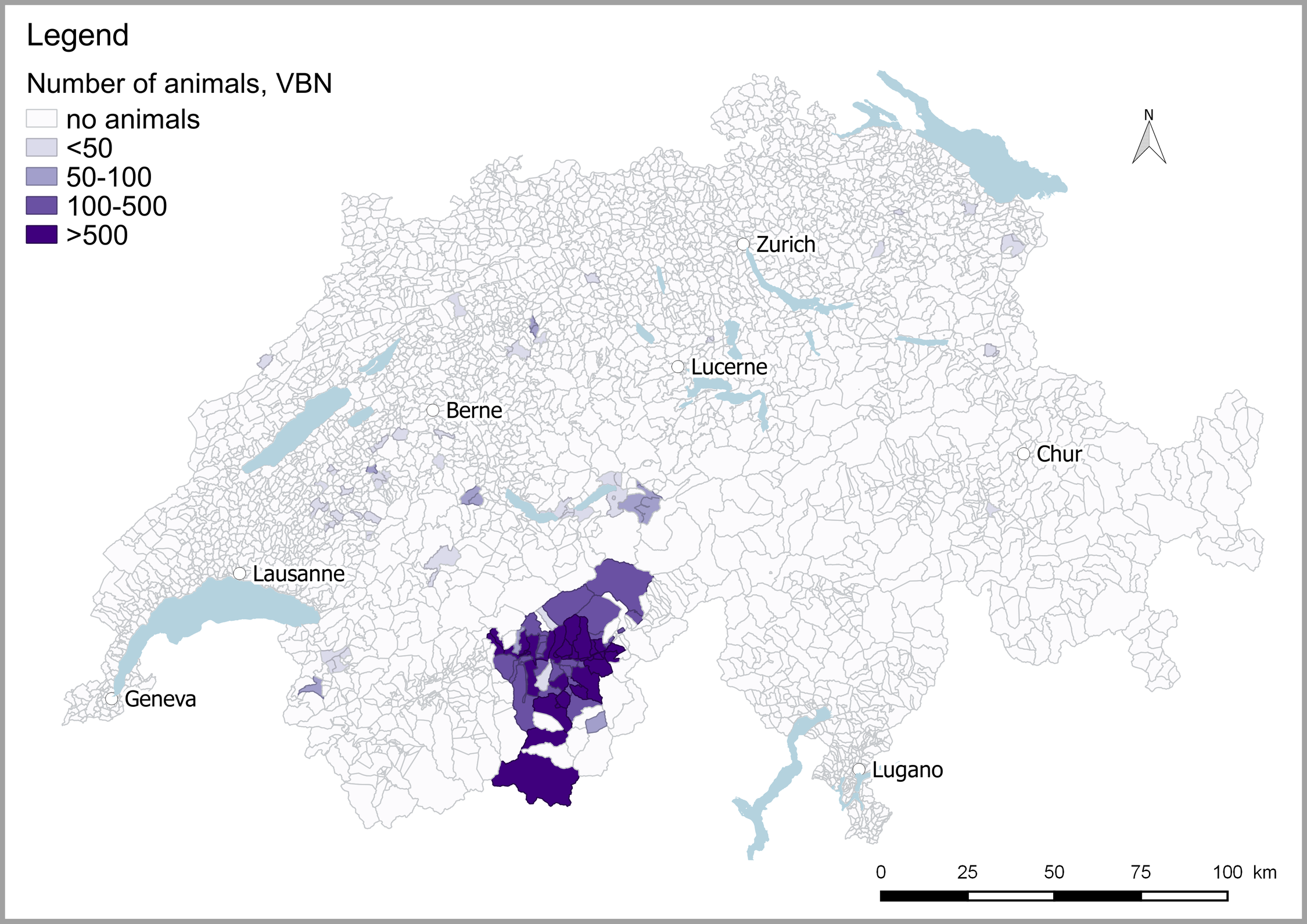

Supplement: S1 Fig — (TIF) [file pone.0176362.s003.tif]

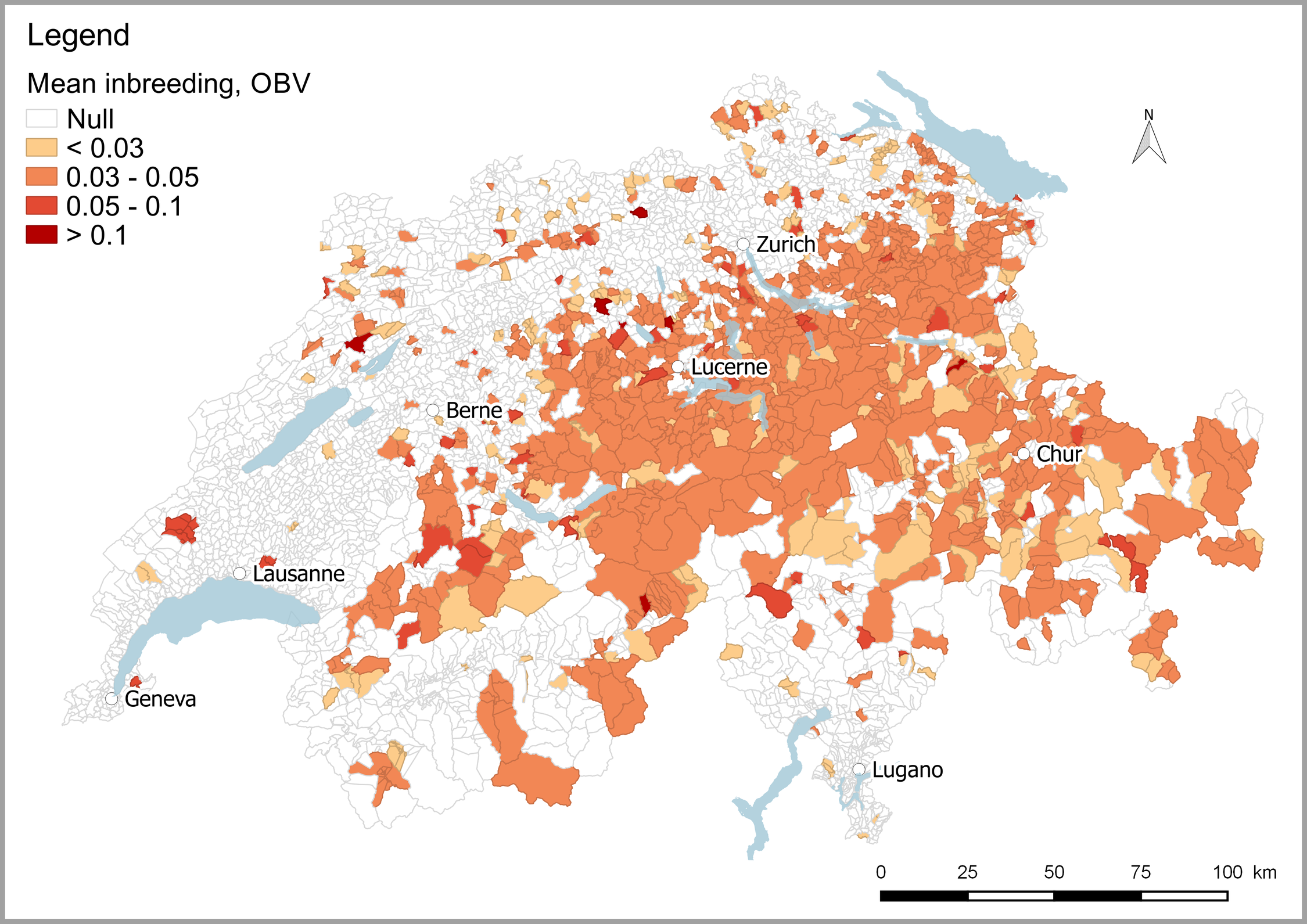

Supplement: S2 Fig — (TIF) [file pone.0176362.s004.tif]

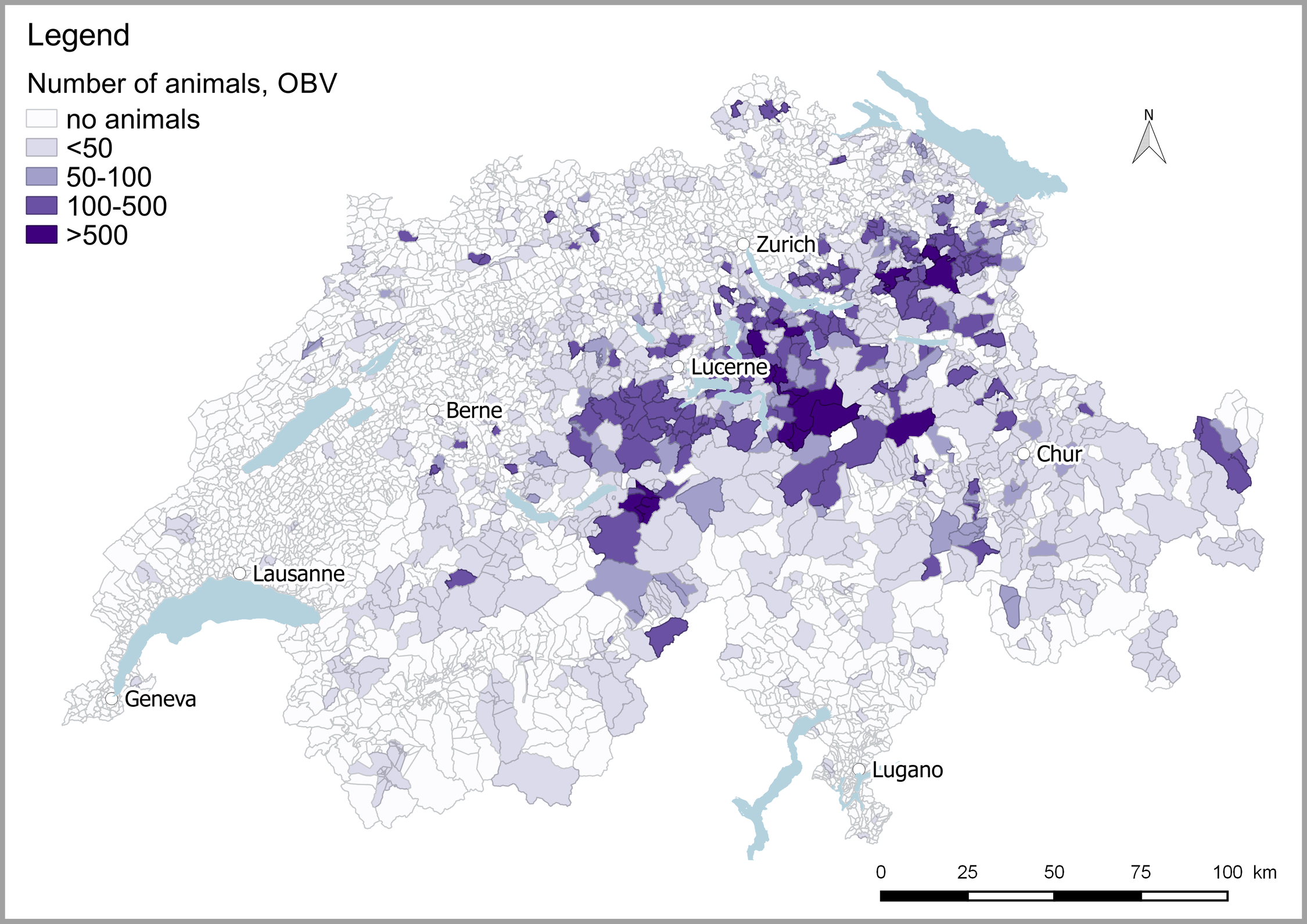

Supplement: S3 Fig — (TIF) [file pone.0176362.s005.tif]

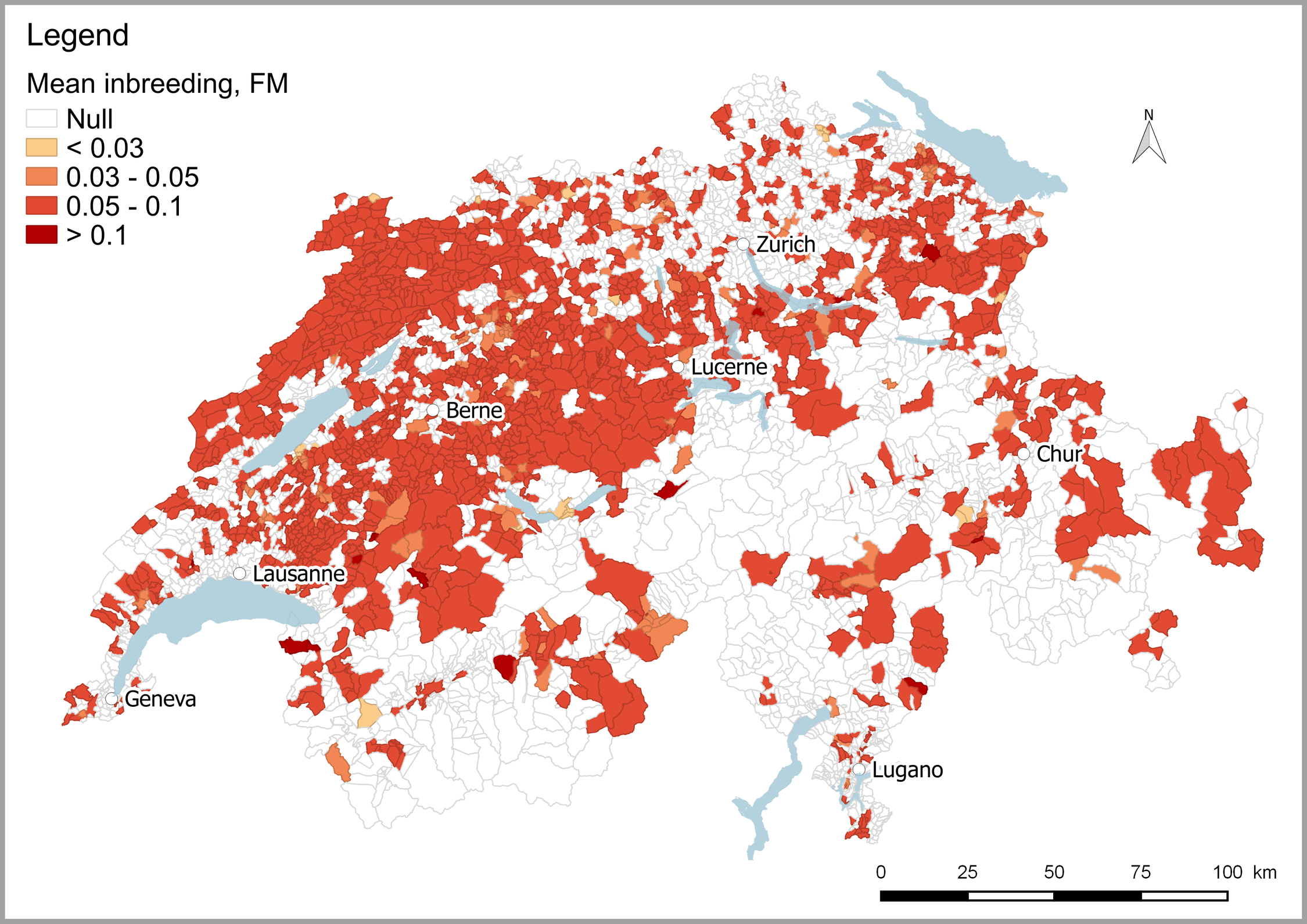

Supplement: S4 Fig — (TIF) [file pone.0176362.s006.tif]

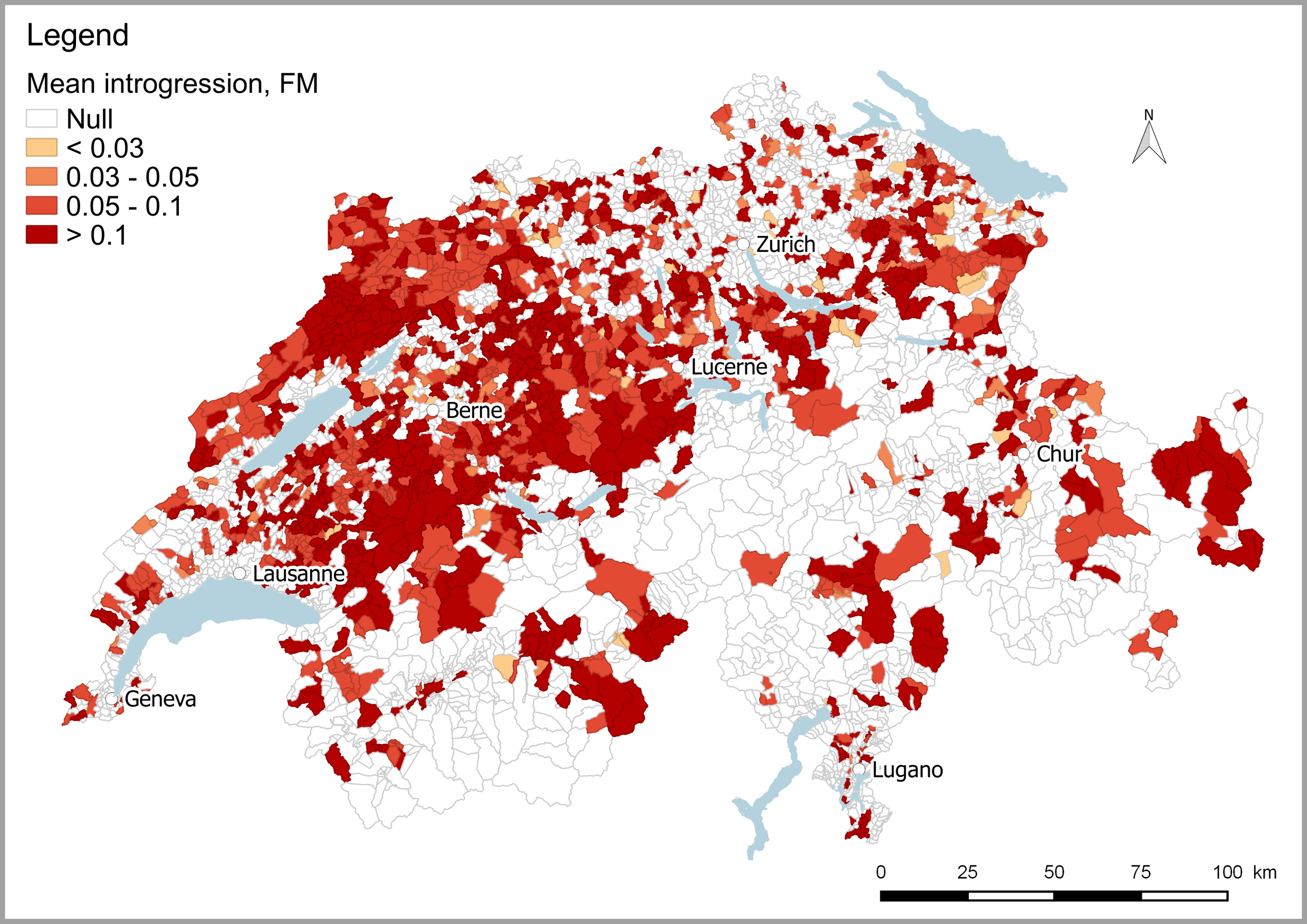

Supplement: S5 Fig — (TIF) [file pone.0176362.s007.tif]

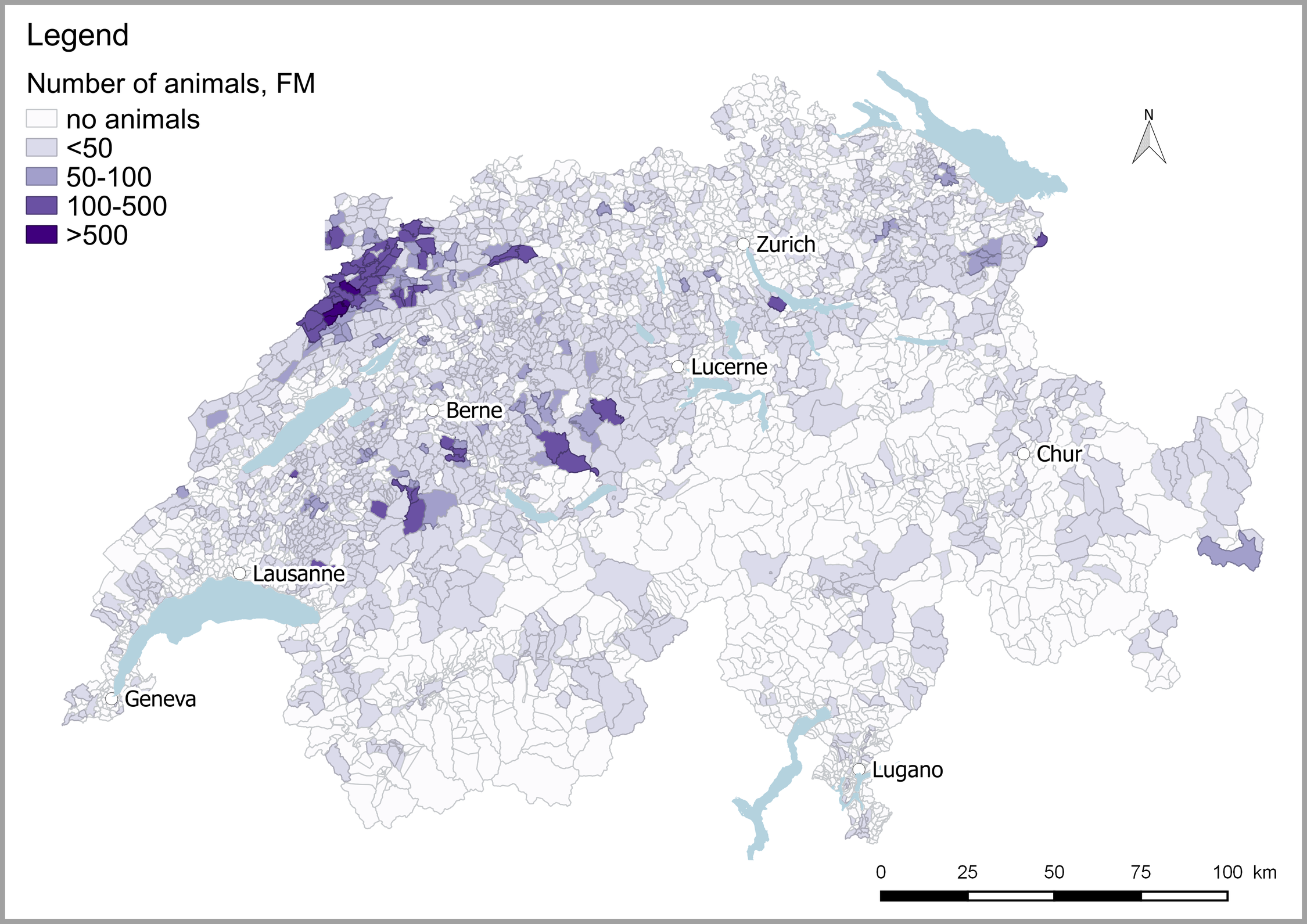

Supplement: S6 Fig — (TIF) [file pone.0176362.s008.tif]
